# Toward the Treatment of Inherited Diseases of the Retina Using CRISPR-Based Gene Editing

**DOI:** 10.3389/fmed.2021.698521

**Published:** 2021-10-01

**Authors:** Jennifer Hernández-Juárez, Genaro Rodríguez-Uribe, Shyamanga Borooah

**Affiliations:** ^1^Jacobs Retina Center, Shiley Eye Institute, University of California San Diego, San Diego, CA, United States; ^2^Medicine and Psychology School, Autonomous University of Baja California, Tijuana, Mexico; ^3^Department of Ocular Genetics and Research, CODET Vision Institute, Tijuana, Mexico

**Keywords:** inherited retinal dystrophies, Leber congenital amaurosis, gene editing, gene therapy, CRISPR-Cas9

## Abstract

Inherited retinal dystrophies [IRDs] are a common cause of severe vision loss resulting from pathogenic genetic variants. The eye is an attractive target organ for testing clinical translational approaches in inherited diseases. This has been demonstrated by the approval of the first gene supplementation therapy to treat an autosomal recessive IRD, RPE65-linked Leber congenital amaurosis (type 2), 4 years ago. However, not all diseases are amenable for treatment using gene supplementation therapy, highlighting the need for alternative strategies to overcome the limitations of this supplementation therapeutic modality. Gene editing has become of increasing interest with the discovery of the CRISPR-Cas9 platform. CRISPR-Cas9 offers several advantages over previous gene editing technologies as it facilitates targeted gene editing in an efficient, specific, and modifiable manner. Progress with CRISPR-Cas9 research now means that gene editing is a feasible strategy for the treatment of IRDs. This review will focus on the background of CRISPR-Cas9 and will stress the differences between gene editing using CRISPR-Cas9 and traditional gene supplementation therapy. Additionally, we will review research that has led to the first CRISPR-Cas9 trial for the treatment of CEP290-linked Leber congenital amaurosis (type 10), as well as outline future directions for CRISPR-Cas9 technology in the treatment of IRDs.

## Introduction

Inherited retinal dystrophies [IRDs] represent a diverse group of rare diseases in which genetic mutations are the principle cause of visual dysfunction (1). The incidence of IRDs is estimated to be 1:2000–1:3000 and IRDs are the leading cause of vision loss in those aged between 15 and 45 years (2–4). IRDs result from pathogenic variants in more than 250 genes expressed mainly in photoreceptors, and to a lesser extent in retinal pigment epithelial cells [RPE] (1, 5–7).

Most IRDs are currently untreatable. This has prompted the development of novel therapeutic strategies. Opportunities for gene or mutation specific clinical translation have been greatly assisted by the increasing availability and reduced costs of high throughput, next-generation sequencing techniques, which use targeted capture and which enable the identification of the molecular cause of ~50–70% IRDs (8–11). Genomic editing approaches have also been greatly facilitated by the discovery and development of the Clustered Regularly Interspaced Short Palindromic Repeats [CRISPR] and the CRISPR-associated genes [Cas]. These achievements have been underscored by the award of the 2020 Nobel Prize in Chemistry to Emmanuelle Charpentier and Jennifer Doudna for the discovery of the CRISPR-Cas9 gene editing system. Although the CRISPR-Cas9 system has been extensively used in basic science and preclinical studies, there has been little work on the *in vivo* application of this editing tool in humans. However, recently, an IRD was chosen as the target for the “first-in-human” *in vivo* clinical trials of CRISPR-Cas9 gene editing, and while these trials raise the possibility of its use in other clinical applications, it faces challenges related to the need for further data to determine the level of efficiency and safety in the clinical setting.

This review will cover the clinical translation of CRISPR-Cas9-based gene editing and will focus on the preclinical work that has led up to and the format of the first human CRISPR-Cas9 gene editing trials. In addition, the review will summarize the current barriers to the wider use of CRISPR-Cas9 gene editing in the clinic and will look to future directions in gene editing to treat IRDs.

## Gene Supplementation Therapy vs. Gene Editing

The eye is particularly suited to clinical translation studies. This is due to its relative immune-privilege, tight blood-ocular barriers (12), and the ability to use non-invasive assessment of retinal structure and function, which facilitates the monitoring of therapeutic efficacy (13–15), and so it is perhaps unsurprising that the eye was amongst the first organs used for gene therapy studies. Furthermore, in the last decade, molecular biology techniques have improved the molecular diagnosis of IRDs. Classical gene therapy consists of gene supplementation, which enables the restoration of defective gene function or the supply of a missing gene by introducing a functional copy of the gene to target cells (5, 13, 16). In 2017, the first ever adeno-associated virus [AAV]-mediated gene supplementation with voretigene neparvovec-rzyl therapy (Luxturna) was approved, firstly in the United States by the FDA and later in Europe by the EMA, to treat Leber congenital amaurosis type 2 [LCA2], the most severe of IRDs. Research in retinal gene therapy has been intensifying lately. Similar efforts are currently ongoing, which are in preclinical and clinical stage, for the treatment of many other IRDs (17), including achromatopsia (*CNGA3* [NCT02610582, NCT02935517, NCT03758404] and *CNGB3* [NCT02599922, NCT03001310]), choroideremia (*CHM*) [NCT02341807, NCT02407678, NCT02671539, NCT03496012 and NCT03507686], retinitis pigmentosa [RP] (*RPGR* [NCT03116113, NCT03252847, NCT03316560, NCT04671433], *MERTK* [NCT01482195], *RLBP1* [NCT03374657] and *PDE6B* [NCT03328130]) and X-linked retinoschisis (*RS1*) [NCT02317887, NCT02416622] (14, 18), with successful proof-of-concept reported in at least 24 genetic forms of disease (1).

AAV is the most common delivery vector chosen for ocular diseases due to its ability to efficiently transduce various retinal cell types *in vivo*, with relatively limited immune reaction and without the need to integrate into host DNA to express genes (Table 1) (19). The lack of integration of AAV leads to a decrease in gene expression over time, however, a single dose of a vector can produce a long-term expression of the transgene, even without transgene integration (17).

**Table 1:** Comparison of gene supplementation and CRISPR-Cas9 genome editing strategies.

**Feature**	**Gene Supplementation**	**CRISPR-Cas9 genome editing**
Can be used to treat autosomal recessive disorders	✓ Although currently restricted by gene size	✓ No restrictions of gene size but limited by gene editing loci
Can be used to treat dominant-negative conditions	✓	✓
Can be used to treat autosomal dominant diseases		✓
Can correct pathogenic variants		✓
Modifies disease without altering the genome	✓	
Causes host immune response	✓	✓
Risk of genotoxicity from overexpression	✓	
Potential off-target effects, such as cleavage or genetic modification of DNA regions other than the intended target site		✓

Although gene supplementation therapy appears to be the best approach for the treatment of autosomal recessive and X-linked forms of IRDs, in which disease is the result of loss-of-function mutations, gene supplementation may not be the optimal solution for counteracting gain-of-function mutations in autosomal dominant IRDs as the pathogenic mutant protein continues to be expressed. In addition, at present, the use of gene supplementation therapy is limited by the packaging capacity of AAV (~4.5 kb). As a result, larger genes, such as *ABCA4*, the most common cause of Stargardt disease and juvenile macular dystrophy, and *USH2A*, the most common cause of adult RP and Usher syndrome, are currently not amenable for all-in-one AAV-mediated approaches. Similarly, *CEP290* (cDNA ~8 kb), whose mutations cause Leber congenital amaurosis type 10 [LCA10], is currently too large for this approach (20). Consequently, gene supplementation approaches have pivoted to delivery alternatives, such as lentiviruses with a packaging capacity of 8–10 kb (i.e., for LCA type 1, 10, or 16) (21–23), and other non-viral vectors including plasmids (24), DNA nanoparticles (25), and antisense oligonucleotides [ASOs] (i.e., for LCA10) (26). These techniques may be less useful for long-term gene therapy as they require repeated injections, but plasmids and nanoparticles may be useful for gene editing requiring shorter expression times.

Many IRDs result from a gene that codes for a structural protein, whose expression must be regulated to be in a proper ratio with the other components to function properly. Therefore, a favorable response to gene supplementation is limited by a narrow range of gene expression, as it is the case for photoreceptors that are sensitive to the amount of transgene expression (22). However, exogenous gene expression may not be regulated similarly to the endogenous gene, since supplementation therapy vectors may include, instead of a cell-specific promoter, a non-specific ubiquitous promoter in the expression cassette, such as the commonly used human cytomegalovirus [CMV] promoter. By improving gene expression, ubiquitous promoters decrease the minimum dose of the viral genome, although this can potentially alter cellular homoeostasis and, in turn, worsen degeneration. For example, Xiong et al. demonstrated photoreceptor toxicity is related to the specificity of the promoter used in AAV vectors for gene supplementation (27).

In order to overcome some of the limitations of gene supplementation therapy, genomic editing approaches have recently become of interest (28). Gene editing uses much of the knowledge gained from gene therapy trials to deliver gene editing machinery to cells, but it offers the potential to permanently treat a variety of mutations through targeted genomic modifications (6).

## Clustered Regularly Interspaced Short Palindromic Repeats

**Figure 1:**
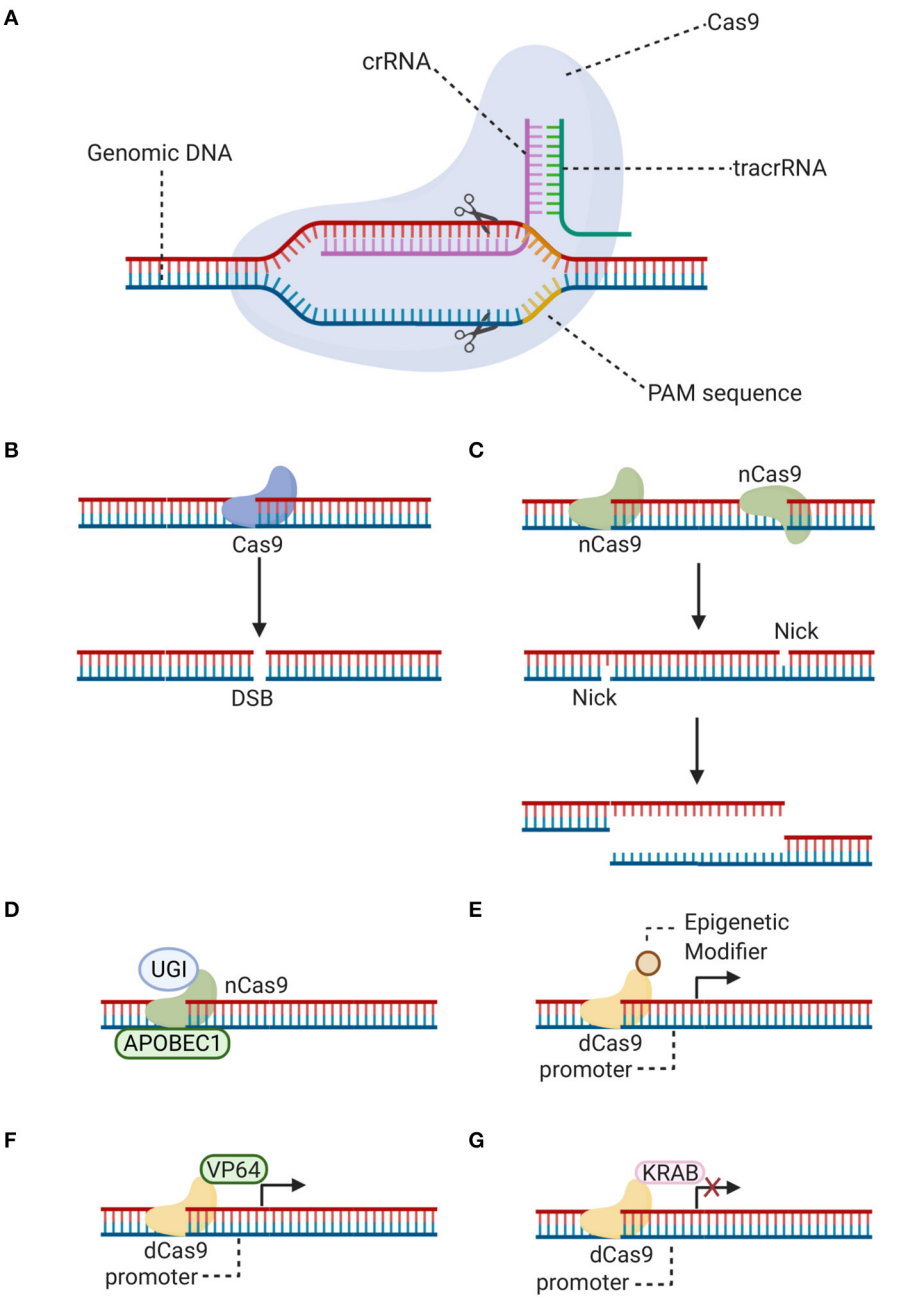
CRISPR-Cas9 editing. **(A)** Schematic representation showing how a double-strand break is generated with the CRISPR-Cas9 system. Specificity is brought about by the singue-guide RNA (fused tracrRNA:crRNA), associated with the Cas9 endonuclease, which recognizes its target nucleotide sequence (protospacer). Additionally, there is a requirement for a protospacer adjacent motif [PAM] downstream of the cleavage site. **(B)** A double-strand break [DSB] can be generated by Cas9 at the target locus, after the single-guide RNA forms a heteroduplex with the protospacer target sequence. **(C)** Alternatively, a double-strand break can be generated by two Cas9 nickases [nCas9], which are mutant variants that create a “nick” on single target DNA strands. Instead of blunt ends, long overhangs are produced at each of the cleaved ends (a staggered double-strand break). **(D)** Cas9 nickase (nCas9) is fused to cytidine deaminase (APOBEC1) and uracil glycosylase inhibitor (UGI) for base editing without double-strand breaks. **(E–G)** Catalytically inactive Cas9 [dCas9] is nuclease-defective but possesses DNA binding ability. dCas9 is fused to epigenetic modifiers such as methyltransferases and acetyltransferases **(E)**, transcriptional activator (VP64) **(F)** or repressor (KRAB) **(G)** domains to achieve targeted gene regulation. Illustration created with BioRender software.

Among the programmable site-specific nucleases for gene editing, CRISPR-Cas9 platform has emerged as an efficient alternative to ZFN and TALEN (17). Jinek et al. demonstrated that the CRISPR-Cas9 immune defense system of *Streptococcus pyogenes* [SpCas9] uses a crRNA and a tracrRNA to direct Cas9 to specific DNA locations and induce a double-strand break [DSB] (Figure 1) (29). This bacterial system has been adapted in the laboratory for genome editing in eukaryotic cells, but both RNA molecules (tracrRNA:crRNA) have been synthetically fused to produce a single-guide RNA [sgRNA], which is equally efficient at binding DNA and guiding Cas9 to specific complementary sequences (29).

CRISPR-Cas9 specificity results from the sgRNA, which requires 20 nucleotides that must match the target sequence in a genomic locus (29, 30). A protospacer-adjacent motif [PAM] is a second requirement of this system. The PAM sequence is positioned immediately downstream of the cleavage site at the 3' end (Figure 1). For example, the PAM sequence 5'-NGG-3' is necessary for SpCas9 enzyme, where N can be any nucleotide and G is a guanine (6, 31). The combination of both, the sgRNA and the PAM site, attracts Cas9 to generate a target-specific DSB (29, 30). This easy-to-construct and highly adaptable gene editing tool has opened up opportunities in ophthalmic translational studies. Nonetheless, *in vivo* use of CRISPR-based therapies is still in its infancy (32). Although the first clinical CRISPR gene editing trial, described in more detail below, represents a huge step forward, concerns remain about the efficacy and general safety associated with this gene editing tool. Attention must be paid in the selected delivery vector, route of administration and issues related to the immunogenic response.

## Mechanisms of Gene Editing

CRISPR-Cas9 generates a DSB, which rapidly stimulates one of two DNA repair pathways: homology-directed repair [HDR], or the more error-prone non-homologous end-joining [NHEJ] (Supplementary Figure 1) (6, 17, 33).

### Non-homologous End Joining

NHEJ is the default method of repair when a DSB is generated by ligation of the two DNA ends at the cleavage site, and it is active during the cell cycle in a variety of adult cells, including proliferating and post-mitotic cells (30, 34, 35). NHEJ repair enzymes (nucleases, polymerases, and ligases) act in a flexible manner, iteratively, randomly, and sometimes independently of one another at each of the two DNA ends (36). As a result, the enzymes restore DNA structure, but usually introducing insertions or deletions [indels] of a few nucleotides at one or both DNA ends, which facilitates end rejoining (35, 36). The indels are generated randomly and may cause frame shifting or exon skipping mutations in protein-coding sequences, which results in premature stop codons for protein synthesis, non-functional proteins, or destruction of messenger RNAs [mRNAs] by nonsense-mediated decay pathway (34). Thus, NHEJ is a preferred tool for gene knockout. As a proof-of-concept for this strategy in IRDs, CRISPR-Cas9 editing approaches have been used to generate NHEJ-mediated disruption of the mutated rhodopsin gene [*RHO*]. RHO protein, which is the most abundant in the disc membranes, is a light receptor densely packed in the outer segments of rod photoceptors and plays a central role in phototransduction. Most of its mutations cause misfolding and aggregation of the apoprotein opsin (37). Moreover, *RHO* mutations are responsible for 20% of dominant RP cases. In the study by Latella et al., the CRISPR-Cas9 approach was used for human *RHO*-specific editing of the most common c.68C>A conversion, upon NHEJ-mediated repair (38). This gain-of-function mutation results in the p.Pro23His [P23H]-RHO mutation in the N-terminal domain, causing destabilization of rod photoreceptor disc membranes (39). Two sgRNAs were designed, targeting the gene and not the mutation, and combined in a single pX330 plasmid expressing SpCas9. The generation of two DSBs excised a DNA fragment at the P23H region in exon 1. Lentiviral-*RHO* transduced HeLa cells were used as an *in vitro* model to validate the efficacy of sgRNA. No cytotoxicity and no significant modifications at predicted off-target sites were noted. For *in vivo* editing, a transgenic mouse model expressing only the P23H-*RHO* mutant allele was used. After subretinal injection, retinas were electroporated to induce plasmid transfection. Indel editing (4–33%) was demonstrated in 9 out of 10 Cas9^+^ tested retinas. The presence of a shorter *RHO* transcript corresponding to the deletion of a fragment of exon 1 was demonstrated by RT-PCR. Substantial decreased expression of mutant RHO protein (56–77%) in photoreceptor cells confirmed the efficacy of this approach (38).

Other studies have reported the selective knockout of mutant *RHO* alleles. In the study by Bakondi et al., transgenic S334ter rats were used as a model of autosomal dominant RP. In the engineered mouse model, the S334ter mutation generates a premature stop codon and consequently protein truncation. Thus, the CRISPR therapy was based on the selective knockout of the mutant *Rho* gene, sparing the wild-type [WT] gene. Using this strategy, the toxic effect of the RHO^S334^ truncated protein was removed, allowing normal RHO^WT^ trafficking to outer segments, photoreceptor survival and visual acuity preservation (40). In the study by Giannelli et al. (39), CRISPR-Cas9 demonstrated its translational potential as it was also efficient for the selective inactivation of the P23H *Rho* mutant allele, while sparing the WT allele. To discriminate between both alleles, the sgRNA seed sequence included the P23H mutation, considering a PAM site that is conserved in the human genome and specific for the SpCas9-VQR variant. After *in vitro* validation of allele-specific targeting in mouse embryonic fibroblasts, the strategy was tested in the retina of newborn *Rho*^WT/P23H^ mice. The intravitreal delivery of dual AAV9-PHP.B synthetic vectors carrying a TetON-inducible promoter for Cas9-VQR and a GFP-sgRNA allowed moderate disruption of mutant alleles (9.6% of photoreceptor cells), without detectable indels at predicted off-target sites. This strategy not only delayed retinal degeneration but also rescued functional activity (39). Recently, a similar approach was used by Patrizi et al. (41). SpCas9 or its VQRHF1 variant were each combined with allele-specific sgRNAs to target the c.1039C>T mutant allele in *RHO* exon 5, while preserving the WT allele. This dominant mutation encodes the p.Pro347Ser substitution in the intracellular C-terminal domain. The *in vitro* characterization of this strategy in engineered Pro347Ser HeLa cells proved its feasibility for discriminating and inactivating the mutant allele, with similar efficacy with either SpCas9 or its VQRHF1 variant. Subsequently, Pro347Ser transgenic mice were used to test the potential to translate previous *in vitro* findings. P7 mice received a single subretinal injection of the CRISPR-Cas9 system, which was packed into dual AAV2/8 vectors containing either SpCas9 or its variant, and their corresponding GFP-tracked sgRNA. Frameshift-promoting indels were the most common modifications in retina cells. It was therefore suggested that the therapeutic benefit of this strategy could derive from the destabilization of the mutant transcript, which decreased significantly (20–60%) in 11 out of 20 retinas. This strategy led to the rescue of retinal function as demonstrated by full field electroretinography and pupillary light responses (41). Taken together, the ability of CRISPR-Cas9 to discriminate among specific alleles *in vivo* makes it a good choice for dominant IRDs.

### Homology-Directed Repair

HDR is a less error-prone repair pathway but occurs much less frequently than NHEJ. HDR naturally occurs in the late S and G2 cell cycle phases, when there is a DNA template that has homology to the region encompassing the DSBs (Supplementary Figure 1). In the gene editing context, a short fragment of single- or double-stranded exogenous DNA can be provided to act as a template (17, 30, 33). The HDR-based strategy allows to knock-in genetic modifications at a certain locus, to replace both loss-of-function and gain-of-function alleles, recovering gene function and eliminating pathogenic effects (42, 43). Additionally, HDR approaches have been extended to progenitor cell types, including embryonic stem [ES] cells and induced pluripotent stem [iPS] cells, encouraging their further development for human and animal modeling in a broad range of genetic conditions, and this could potentially be an approach for *ex vivo* correction of mutations prior to transplant back into patients (Supplementary Figure 2) (33).

HDR-mediated repair was previously considered restricted to dividing cells, representing a significant limitation for *in vivo* applications, such as correcting post-mitotic photoreceptors. But later, it was found that CRISPR-Cas9-mediated HDR can be effective in editing post-mitotic cells, such as neurons, although with reduced efficiency (44). The first proof-of-principle of this strategy in the visual system was demonstrated using CRISPR-Cas9-mediated HDR to enable editing of the mutated *Pde6b* gene in the retina of the “rodless” [rd1] mouse, the most studied model of RP. A single-stranded oligodeoxynucleotide donor template was used to repair the nonsense mutation Y347X (C-to-A) in exon 7 that causes protein truncation. The CRISPR-Cas9 machinery and the *Pde6b* donor template were co-injected into FVB/N zygotes, generating 11 founders. HDR events occurred in four animals, but the donor template was precisely incorporated only in two animals in ~36% and 19% of photoreceptors. These percentages were enough for a sustained restoration of ERG responses, for at least 2 months, although these responses were not comparable to the wild-type responses. Retinal structure was also partially restored, as there was a partial rescue of the outer nuclear layer [ONL] (76 and 50% of normal thickness), whereas the ONL was not detectable in rd1 mice (45). In another proof-of-principle, HDR rescued the phenotype of the rd12 mouse model of human LCA, which carries a disease-associated mutation in *Rpe65*. CRISPR-Cas9 was used to induce DNA cleavage and HDR-mediated editing to correct the nonsense mutation causing a premature stop codon in *Rpe65*. Treatment consisted in the subretinal injection into 3-week-old rd12 mice of a dual AAV system expressing CRISPR-Cas9, a sgRNA targeting the C-to-T nonsense mutation, and a single-stranded oligodeoxynucleotide used as an *Rpe65* donor sequence. After 4 weeks of AAV treatment, HDR events were induced in the RPE, but not in the retina. The frequency of gene correction was ~3%, considering HDR and in-frame NHEJ. HDR events induced precise correction of the mutation at a frequency of ~1.2%, thus the resulting protein sequences were identical to wild-type *Rpe65*, whereas the NHEJ events induced an in-frame deletion of the pathogenic stop codon in *Rpe65* at a frequency of ~1.6%. The treatment also led to a sustained recovery of Rpe65 expression, retinal function recovery (with levels up to 21 and 40% for a- and b-wave amplitudes in ERG responses), and maintenance of retinal thickness (46). These results confirmed that CRISPR-Cas9–mediated HDR can be used in post-mitotic cells.

### Homology Independent Targeted Insertion

A new strategy emerged since HDR-based approaches are limited by low efficiency in most primary cell types, and HDR pathway is infrequent in non-dividing cells. Homology independent targeted insertion [HITI] is a modification of the gene knockout strategy, enabling the integration of exogenous DNA, via NHEJ pathway, in non-dividing cells (Supplementary Figure 1) (47). Suzuki et al. developed this strategy to integrate, in a site-specific manner, a corrective donor DNA, by designing a plasmid flanked by Cas9 cleavage site sequences in reverse orientation to those found at the genomic locus. Hence, Cas9 cleaves both the genome and the donor plasmid. Once the donor DNA is inserted in the desired orientation, the Cas9 target sequence is disrupted and there is no further cleavage. HITI demonstrated to be feasible for targeted knock-in in dividing cell lines, with an efficiency 10 times higher than that for HDR, and predominantly error-free (42, 43). As a proof-of-concept for *in vivo* application, HITI demonstrated its efficiency in non-dividing cells using the Royal College of Surgeons rat model of autosomal recessive RP, caused by ~2 kb deletion of the *Mertk* gene, from intron 1 to exon 2, leading to dysfunctional RPE and retinal degeneration. The HITI system and the sgRNA were AAV-delivered via subretinal injection. As the full exon 2 was knock-in into the genome, *Mertk* mRNA and protein levels increased, and there were considerable improvements in retinal morphology and rod-cone responses; however, the rescue was not enough to fully restore vision (42). In another study of Llado et al., HEK293 cells were co-transfected with a plasmid expressing mouse *Rho*, another plasmid expressing Cas9-GFP and a sgRNA targeting the first exon of mouse *Rho* (mRHO), and a plasmid carrying dsRED as the donor DNA. As a result, Cas9 cut the target locus and the donor DNA. Precise integration occurred in ~70% of transfected cells. Also, 2.5 × 10^9^ genome copies of each of two AAV8 vectors encoding Cas9 and the donor DNA were injected subretinally into C57BL/6 mice. One month after treatment, up to 9% of dsRED was efficiently integrated into rod photoreceptors, but only when the donor DNA was co-administered with the mRho sgRNA. Lastly, 2 × 10^11^ genome copies of each AAV8 were injected subretinally into pigs (using a pig-specific sgRNA and donor DNA-flanking region) and after a month, dsRED-positive photoreceptors were observed (48). Noting that HITI is feasible for *in vivo* genome editing in the retina of mice and large animal models, the same group is adapting this system to integrate a functional copy of the RHO coding sequence into a mouse model of autosomal dominant RP to get its knockout and replacement at the same time (48). Still, to translate HITI technology into clinical treatments, higher gene-correction efficiencies will be required which may be achieved with improved Cas9 orthologs, such as high fidelity Cas9 (43).

### Base Excision Repair Mechanism

One of the concerns of traditional CRISPR-Cas9 gene editing is the generation of unpredictable indels as a result of the NHEJ repair after the generation of DSBs, which are then permanently introduced into the genome. A further, promising alternative traditional CRISPR-mediated approach of genome editing allows the direct and irreversible base conversion, without inducing DSBs or exploiting HDR. To enable programmable installation of four transition mutations (C to T, A to G, T to C, and G to A) in genomic DNA, Liu's group developed a fusion protein comprising a catalytically inactive Cas9 [dCas9] or a Cas9-D10A nickase, the rat cytidine deaminase APOBEC1 or adenine deaminase, and an inhibitor of the cellular base excision repair mechanism such as uracil glycosylase inhibitor (Figure 1). The resulting base editors showed high base editing efficiency in multiple cell lines and low indel frequency (49, 50). Additional base editors have been engineered using Cas orthologs or variants as effectors, with alternative PAM sequences, to increase their editing repertoire and efficiency (49, 51). Overall, this approach is highly promising, as it allows the potential for precise gene editing in post-mitotic cells without the variability of indel formation. Previously, base editing was limited to correction of point mutations. Although ~50% of human pathogenic variants result from point mutations, a large number of indels, duplications, copy number loss and gain variants also result in disease (52). Of note, the scope for editing variants other than point mutations with base editors has increased more recently, a “search-and-replace” genome editing approach has been proposed to mediate all possible base-to-base transitions, targeted insertions and deletions with small numbers of bases, which is predicted to correct up to 89% of genetic variants associated with human diseases (52).

### Target-Specific Epigenetic Editing

In addition, although this review has focused on the use of CRISPR-Cas9 for genome editing, recent studies have highlighted its utility for target-specific epigenetic regulation, in a reversible way. To this end, CRISPR uses dCas9, which lacks endonuclease activity, and a customized sgRNA (53). The dCas9-sgRNA complex, in combination with transcriptional effectors, can be used for gene repression [CRISPRi] or gene activation [CRISPRa] (54), by repositioning regulators of transcription, modifiers of histone state and DNA methylation, and ncRNAs (Figure 1) (55). These strategies pave the way in the research of new therapeutic options for curing diseases.

The CRISPRi mechanism is based on steric hindrance of dCas9-sgRNA complex at the promoter or coding region of the target gene. DNA binding of the dCas9-sgRNA complex on its own blocks access of transcription factors or the transcriptional machinery preventing transcription initiation or elongation, although its repression capacity is increased when dCas9 is fused to repressor domains (53, 56). CRISPRi has a strong safety profile, as off-target repression is extremely rare, and the effects are completely reversed upon degradation of dCas9 or sgRNA (54, 56–58). CRISPRi could be used to regulate the expression of genes involved in IRDs, especially those that are difficult to edit. Since its activity is highly sensitive to sgRNA-DNA mismatches, it can accurately discriminate and silence pathogenic alleles (59). Thus, CRISPRi could be viable to treat gain-of-function mutations in IRDs, such as *RHO* or *PRPH2* mutations in autosomal dominant RP. In Moreno et al. study (58), dCas9 was coupled to diverse transcriptional effectors, with activating or repressing activity. After validation of their system *in vitro*, it was tested in rd10 mice, a model of autosomal recessive RP carrying a mutation in the *Pde6*β gene that leads to rod degeneration. To this end, the KRAB repressor domain was fused to dCas9 (Figure 1), resulting a more effective inhibitor than dCas9 alone, and used to silence the *Nrl* gene, a master regulator of rod photoreceptor differentiation. The simultaneous downregulation of *Nrl* and upregulation of cone-specific genes in treated mice prevented vision loss, as a result of rod reprogramming into a more cone-like phenotype, with a thicker ONL, and improved visual function indicated by higher visual acuity. It should be noted that the significant rescue of cone function may reduce rod function and number, which could lead to night blindness. However, the potential benefits would outweigh this acceptable risk (58).

CRISPR is now also being used to target mRNAs by another CRISPR associated protein, Cas13 (60). Taken all together, this highlights the continued development of the CRISPR-Cas system.

## Barriers to the Use of CRISPR-Cas9 in the Clinic

In a similar manner to gene supplementation therapy, the most common method for CRISPR-Cas9 delivery is with AAV vectors. AAV vectors possess a variety of assets for use in human delivery, including high overall safety. They infect a broad range of host cells, including both dividing and non-dividing cells. The viral genome does not integrate into the host genome, minimizing the risk of insertional mutagenesis. Another advantage of AAV vectors is the availability of at least 13 naturally occurring serotypes that differ in the efficiency and/or specificity of gene transfer to cell and tissue types, including retinal cells (61). In addition, AAV pseudotyping, which consists of packaging the genome of one serotype into capsid proteins of another serotype, impacts on cell tropism by enabling an even more specific targeting toward specific cell types, as in the case of the AAV2/5 serotype which transduces neurons more efficiently than the AAV2 serotype. Also, to increase the efficiency of transduction, hybrid AAV serotypes have been engineered by packaging the genome of one serotype into a mixture of capsid proteins from multiple serotypes, such as the AAV-DJ serotype that involves a mixture of capsid proteins from eight different serotypes (62).

AAVs were initially chosen as they generated a lower immune response than other virus vectors such as adenovirus, within the eye (63). However, there is increasing evidence that the temporary increased immune response to AAV prevents gene expression and, as a result, this is certain to also play a part in CRISPR-Cas9 expression. Increasing evidence points to a reaction to the viral capsid rather than by the gene expressed (19, 64). Of note, anti-Cas9 antibodies and T cells against the most commonly used Cas9 orthologs have been found in the serum of healthy individuals, presumably as these nucleases are derived from frequent pathogens in humans (65). Pre-existing immunity could limit the outcome of therapeutic AAV-CRISPR-Cas9, as it was observed in mouse models that after 6–12 weeks of efficient gene editing, the number of edited cells decreased dramatically due to pre-immunity (66). However, the immune response may be less of a barrier to CRISPR-Cas9 gene editing as only a few days of high gene expression will likely be required to generate enough CRISPR-Cas9 to perform enough editing with a functional benefit (67). Evidence for this comes from studies in P12 mice targeting *Vegfr2* in a mouse model of choroidal neovascularization by Huang et al. (68). Using AAV1-SpCas9 driven by a CMV promoter, 5 days after intravitreal injection, sequencing confirmed about 2% of indels around the PAM. Western blots retinal lysates also demonstrated a reduction of ~30% in VEGFR2 and there were significantly fewer preretinal tufts and significantly more avascular areas, suggesting a functional benefit (68). Even more promising, Kim et al. administered ribonucleoproteins and a sgRNA by subretinal injection to target *VegfA* and found a 25 ± 3% indel frequency in RPE cells only 3 days after injection (69). Besides, these studies showing efficacy in the targeting of *VEGF-A* and *VEGF* receptor genes in models of neovascularization lead to potential treatments for retinal diseases in which the pathology is primarily VEGF-driven, e.g., age-related macular degeneration or diabetic retinopathy, which are not IRDs but rather multifactorial diseases in which progressive visual impairment occurs, or lesions such as macular edema and ischemic retinal vein occlusions.

The immune response is particularly dependent on the route of administration, with intravitreal administration inducing a stronger immune response than subretinal delivery (70). This has hampered efforts for pan-retinal delivery of AAV. This may be especially limiting for the use of CRISPR-Cas9 to treat IRDs. The majority of IRDs, including RP, are pan-retinal. Large subretinal blebs carry the risk of macular hole formation. A way around intravitreal immune uses multiple smaller subretinal blebs targeted to regions with the target cell. However, the number of blebs and CRISPR-Cas9 will have to be carefully titrated to induce a functional effect as all target cells are unlikely to be treated in these smaller subretinal blebs due to *in vivo* CRISPR-Cas9 editing efficiency, reducing the total area of functional cells after treatment. Another approach may be to treat areas multiple times. As CRISPR-Cas9 will only target unedited cells, multiple treatments to the same region may increase the total number of edited cells. It is hoped that problems concerning intravitreal immune response will be solved with the next-generation of evolved AAVs, which are also able to reliably penetrate the outer retina thus removing the need for multiple subretinal injections (https://www.4dmoleculartherapeutics.com).

As a result of the potential problems with AAV immune response reducing gene expression, non-viral vector delivery methods are being investigated. Alternative approaches harness the potential of non-viral vectors (71), including lipids (72), polymers (73), and nanoparticles (74) for carrying Cas9, its sgRNA, and donor DNA. Although there has been substantial progress made for CRISPR-Cas9-mediated gene knockout (75, 76) and knockdown (77) using non-viral vectors along the NHEJ pathway, relatively limited results have been obtained for CRISPR-Cas9-mediated knock-in (78, 79), especially for the integration of a full-length gene. Chou et al. employed supramolecular nanoparticle [SMNP] vectors for co-delivery of DNA plasmids, CRISPR-Cas9 and the *Retinoschisin 1 [RS1]* gene, using the HITI strategy (80). These SMNP vectors were employed for CRISPR-Cas9 knock-in of *RS1/GFP* genes into the mouse Rosa26 safe-harbor site *in vitro* and *in vivo*. The *in vivo* study used an intravitreal injection approach, providing two SMNP vectors into the mouse eyes. The gene was expressed in the retinas, demonstrating effective knock-in of *RS1/GFP* gene (80). This proof-of-concept study highlights the potential of the combined use of the SMNP vectors with the HITI strategy to achieve *in vivo* CRISPR-Cas9-mediated knock-in of a therapeutic gene. CRISPR-Cas9-mediated knock-in of the *RS1* gene in the retinas of X-linked retinoschisis patients, via either intravitreal or subretinal injection of the SMNPs, would offer a novel therapeutic solution which may circumvent the immune system activation associated with viral vectors.

Long-term Cas9 expression in post-mitotic cells, raises concerns about the likelihood of oncogenicity, with the development of off-target mutations and their potential harmful effects, for example chromosomal translocations, as a result of the induction of DSBs (81, 82). So, one of the potential benefits of reduced long-term Cas9 expression may be the reduction of this risk.

Another limitation of AAVs is the restrictive cargo capacity (~4.5 kb). This presents an obstacle for packaging the SpCas9 (4.2 kb) and its sgRNA in a single vector; leaving little room for customized expression and control elements (83). Recent developments have overcome this obstacle. SpCas9 can be split into two fragments, enhancing its compatibility with AAV size capacity (61), which can form a functional full-length nuclease when reassembled in the cell (61, 84). Split-Cas9 maintains the cleavage activity for genome editing, albeit at a reduced level relative to native Cas9 (85) since co-delivery of two AAV vectors is less efficient than the delivery of a single AAV vector *in vivo* (86).

Recently, Cas9 has been discovered in other bacteria. *Staphylococcus aureus* Cas9 [SaCas9] was found to be capable of *in vivo* genome editing with similar levels of efficiency to SpCas9 (83). SaCas9 coding sequence (3.16 kb) is more than 1 kb shorter than SpCas9, so SaCas9 and its sgRNA can be packaged into a single AAV vector (83, 87). Since the discovery of SaCas9, other Cas9 orthologs and variants have been discovered or generated that are not only small enough to be packaged in a single AAV, but also provide other targets in the genome with the variety of PAM sites and help overcome Cas9 size as a barrier to clinical translation (86, 88, 89). For example, the variant KKH SaCas9 that requires the 5'-NNNRRT-3' PAM site for efficient genome editing not only expands the selection of target sequences, but also increases the targeting range of SaCas9 to 2-fold or more (88). Also, one of the smallest Cas9 orthologs derived from *Campylobacter jejuni* [CjCas9] (2.9 kb) induces targeted editing with high specificity *in vitro* and *in vivo*, with frequencies of indel formation comparable to SaCas9 (86). Besides, its PAM sequence (5'-NNNNACAC-3') increase the options of target sequences for *in vivo* genome editing (86). Of note, non-viral vectors are another attractive option as they have no size limitations, however thus far, none have been tested in clinical trials of retinal diseases (20).

Another current challenge is Cas9 specificity. One of the concerns is the generation of off-target editing, which can be potentially deleterious or, in the worst instances, lead to changes in gene expression and tumor formation. This issue is particularly important as off-target editing increases with long-term Cas9 expression (17). Recent efforts have focused on finding CRISPR-Cas9 orthologs or variants, with a high on-target activity but minimal off-target effects (90, 91). High fidelity SpCas9 variants, such as enhanced specificity SpCas9 [eSpCas9(1.1)] (92), Cas9-High Fidelity [SpCas9-HF1] (93), HypaCas9 (94), and SniperCas9 (95) were developed to decrease non-specific interactions with DNA and to diminish the frequency of off-target mutations.

In another approach to increase specificity, Cas9 endonucleases have been converted into nickase mutants [Cas9n] (96) by inactivation of one of its two conserved nuclease domains. Using this method, two nickases must bind nearby to opposite DNA strands, guided by two different sgRNAs, to generate a DSB, thus increasing specificity without giving up efficiency (Figure 1) (97). Another strategy to increase specificity and to evade immune response consists of a reduction of long-term Cas9 exposure by using a self-limiting Cas9 (98, 99). However, the insertion of an additional sgRNA targeting Cas9 itself into a gene editing vector carries the risk of additional off-target effects and a decrease in editing efficiency compared to conventional CRISPR (100). To optimize this strategy, one or two recognition sites for the sgRNAs designed to target a gene sequence can be added into the plasmid, so that the sgRNAs will not only edit the target locus, but also cleave the Cas9 plasmid and, in doing so, prevent long-term Cas9 expression without increasing the risk of off-target editing (100).

As previously mentioned, DSBs generated by Cas9 and a sgRNA at a specific target sequence are repaired by either endogenous DNA repair pathway, however the results of the repair events are not predictable. Point mutations, random indels and deletions extending over many kilobases can be introduced as a result of imprecise NHEJ. Also, the targeted locus can be restored using a template for HDR, yet a major challenge consists in achieving its precise integration. Deep profiling reveals that there are heterogeneous outcomes in CRISPR knock-in experiments, varying between cell-types or the form of DNA donor template used. In addition to the expected integration of donor DNA, which accounts for a minority of events in most cases, the delivery of new DNA may also generate indels and mis-integrations events, including incomplete or incorrect insertions, rearranged or concatemerized donor integration, or extended integration of plasmid backbone sequence (101). An unforeseen risk of using CRISPR-Cas9 for therapeutics has been addressed in Brunner's work, showing that Cas9-mediated editing in *Drosophila* often leads to germline-transmitted recombination of genetic chromosome arms (39% of CRISPR-Cas9 events), with no indels in most of the cases (102).

## Pathway to the Clinic for IRDs

**Figure 2:**
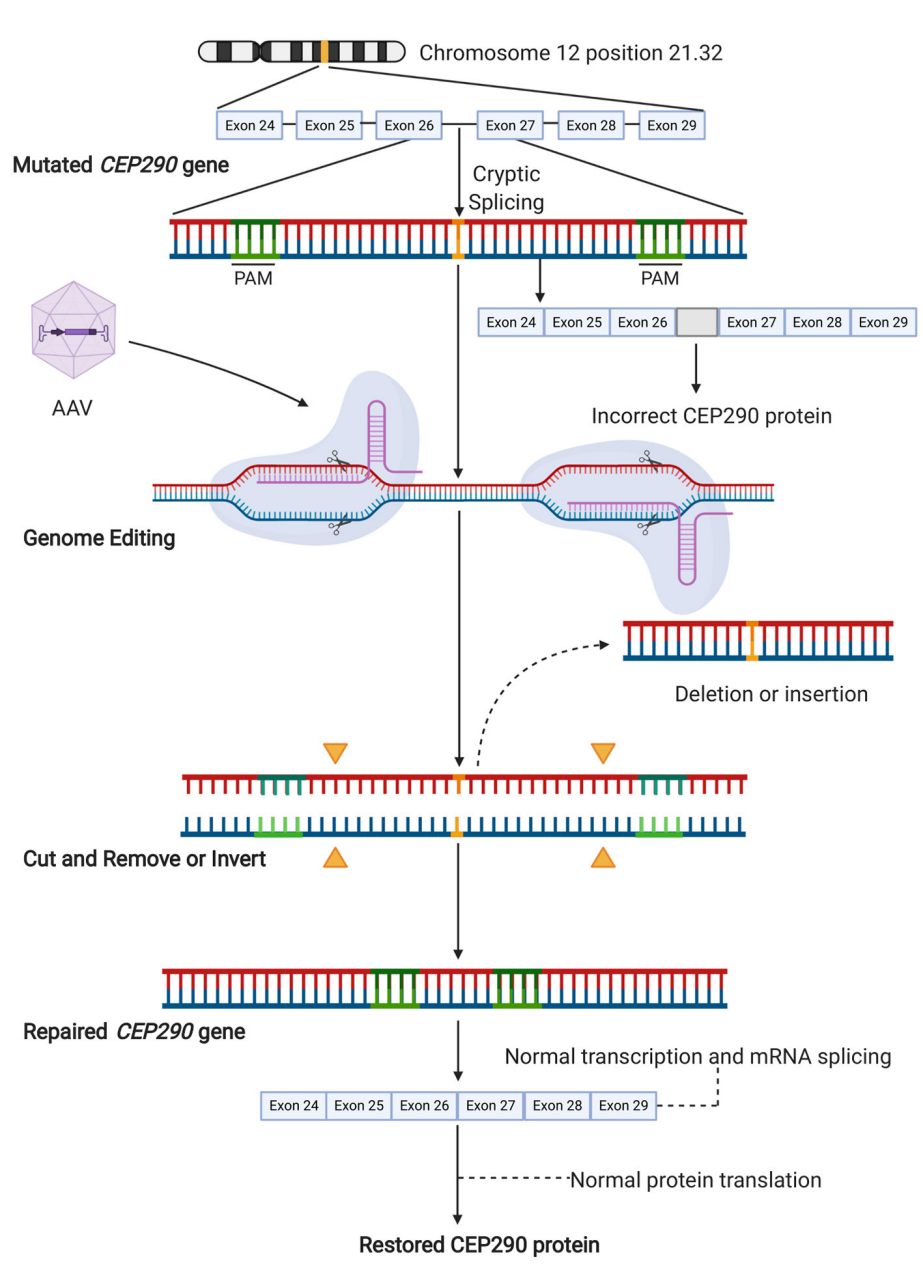
Gene editing approach targeting the cryptic splice site in *CEP290*. The pathogenic variant c.2991+1655A>G leads to the generation of a cryptic splice site in the intron between exon 26 and 27. This variant results in the translation of a non-functional mutant protein. Targeted gene editing on each side of the cryptic splice site aims to the removal of this mutation. Successful editing results in either deletion of the DNA segment containing the mutation or its inversion, which similarly results in the loss of the cryptic splice site. This enables the normal translation of functional CEP290 from wild-type full-length mRNA. Illustration created with Biorender software.

LCA10 is a rare condition that causes photoreceptor dysfunction, degeneration, and severe visual impairment from early infancy. LCA10 is an autosomal recessive disorder caused by mutations in the *centrosomal protein 290 kDa* [*CEP290*] gene (67, 82, 103), which makes it an important therapeutic target. CEP290 is a vital structural and regulatory protein expressed in the transition zone of the connecting cilium, including the photoreceptor cilia, with a significant role in regulating both ciliogenesis and ciliary trafficking (22, 104, 105). Although CEP290-associated LCA10 patients retain some cone photoreceptors in the central foveal region, these photoreceptors have abnormal inner and outer segments leading to, in most cases, significant vision loss (22, 106). The most frequent mutation associated with LCA10 is c.2991+1655A>G (intervening sequence in intron 26 [IVS26]) in *CEP290*. The resulting mRNA encodes a premature stop codon (p.C998X) that generates a truncated protein (Figure 2), retaining only partial activity (67, 82, 103).

The large size of the *CEP290* gene (coding sequence of ~8 kb) precludes AAV-mediated gene supplementation therapy. Also, in many retinal degenerative conditions related to defects in protein trafficking or cilia function, the cell tolerates only a certain amount of protein product (20), as a previous study demonstrated that the alternative lentiviral-mediated CEP290 overexpression results in photoreceptor cytotoxicity (22), which also makes gene supplementation unattractive. Additionally, there are at least 11 transcripts for *CEP290*, most of which encode proteins with unknown tissue expression and function. Therefore, it would be preferable to restore the normal configuration of *CEP290* by correcting its aberrant splicing (107). Ruan et al. (67) proposed a CRISPR-Cas9-based approach for the treatment of IVS26-related LCA10. Their strategy excised the intronic fragment containing the IVS26 splice mutation in *CEP290*. The authors first developed an *in vitro* model of LCA10 using CRISPR-Cas9-mediated HDR to edit in the *CEP290* mutation into human HEK293FT cells. Analyses confirmed that the efficiency of targeted IVS26 deletion was 60–70% in mutant cells, resulting in an increase in normal *CEP290* mRNA and full-length CEP290 protein. In addition, there was no detectable off-target editing. The authors used wild-type mice (C57BL/6J) to test the efficacy of *in vivo* retinal editing with CRISPR-Cas9, using a pair of sgRNAs and a dual AAV5 system (67). As there were no animal models expressing the IVS26 mutation, sgRNAs were designed to target murine intron 25, which is homologous to intron 26 of human *CEP290* (108). The dual vectors, co-delivered by subretinal injection, demonstrated successful editing as 7.5–26.4% of sequences from the retina had truncated DNA (67).

More recently, Maeder et al. assessed the specificity and tolerability of their construct named EDIT-101, which included a pair of highly specific sgRNAs to create two DSBs on each side of the IV26 mutation of *CEP290*, along with SaCas9 under the control of a photoreceptor-specific GRK1 promoter, packaged into an AAV5 vector, in order to remove the cryptic splice site and restore normal splicing. A retinal explant culture system using control *post-mortem* retina was developed to demonstrate the construct efficacy in mature human photoreceptors. Although the spectrum of CRISPR-Cas editing events in the photoreceptors (average editing rate of ~42%) included small indels, large deletions, sequence inversions, and insertions of AAV vector sequences, productive *CEP290* edits (~17%) occurred with large deletions or inversions of the intervening sequence, which corrected the splicing defect of *CEP290*, and thereby restored its normal mRNA and protein expression (82). Subsequently, HuCEP290 mice (human *CEP290* IVS26 knock-in mouse model) were treated with two different dose concentrations of the EDIT-101 construct to confirm the on-target editing *in vivo* in a humanized system. Based on published clinical data, the authors considered that the functional rescue of at least 10% of foveal cones was necessary to achieve therapeutic efficacy (109). Accordingly, 94% or more of treated eyes reached the minimal productive *CEP290* editing rate. Their results suggested that EDIT-101 at a dose ranging from 3 × 10^11^ to 3 × 10^12^ vgml^−1^ may lead to clinical benefit (82). Since mouse retina not only lack macula but also 97% of their photoreceptor cells are rods (110), Cynomolgus monkeys were used as a non-human primate [NHP] model to determine the level of productive editing in a retina anatomically similar to that of humans. For this purpose, AAV5 NHP vectors were constructed similar to EDIT-101 and delivered via subretinal injections within the perifoveal region of NHPs. *CEP290* editing rates of 16 and 28% were reached, which exceeded the minimum predicted therapeutic threshold, using vector concentrations of 7 × 10^11^ and 1 × 10^12^ vgml^−1^, respectively. These findings were similar to those for mice, thus supporting dose extrapolation to the first-in-human trial (82).

In early 2020, an open-label, single ascending dose study started to enroll LCA10 patients to test the CRISPR-Cas9 gene editing method to correct the disease [NCT03872479] delivering the same construct, EDIT-101 (Figure 2), by subretinal injection. The study will enroll up to 18 participants aged 3 years or older, who harbor at least one mutation including the c.2991+1655A>G in *CEP290*, and with vision worse than 0.4 LogMAR (Snellen equivalent 20/50). Participants will receive a single dose of EDIT-101. Up to 5 cohorts across 3 doses will be enrolled in this study. Primary outcome measures will be identified over a 1 year time frame and will include primary outcome measures of frequency of adverse and toxic events with secondary outcome measures, including changes in: visual mobility course score, LogMAR measurement of BCVA, pupillary response, dark adapted visual sensitivity using full field light sensitivity threshold testing, macular thickness, contrast sensitivity, macular sensitivity measured by microperimetry, kinetic perimetry, color vision and quality of life scores. This phase I study is expected to be completed in 2024. The results obtained from this study will be indicative of how applicable CRISPR-Cas treatment may be for other IRDs.

Furthermore, the use of CRISPR-Cas9 in somatic therapies and tissue engineering is likely to increase. For example, in a recent research CRISPR-Cas9 editing was used in the production of retinal cells derived from human ES cells for replacement therapy. The editing consisted in the knockout of *beta-2-microglobulin* [*B2M*], of *class II major histocompatibility complex transactivator* [*CIITA*] or of both, for the generation of RPE cells lacking surface presentation of human leukocyte antigen class I [HLA-I] or/and HLA-II. These modifications helped cells to evade the immune system and reduce their rejection in a rabbit model (111).

## Future Perspectives in the Clinic

Successful demonstration of safety in the first trial with CRISPR-Cas9 is likely to lead to other studies targeting a wider range of diseases. Treatment trials for other IRDs are already in the research development pipeline, including diseases caused by *RP4* gene mutations (RP4) and the more common Usher syndrome resulting from *USH2A* gene mutations (EDIT-102) (112–114).

At present, in our view, CRISPR-Cas9 is likely to only fill a niche role in the treatment of IRDs. The currently variability of editing efficiencies at each locus, potential for off-target changes with each new construct, and the continued concerns around permanently editing the genome in an unpredictable manner with knockout strategies will likely prevent its wholesale use. The variability at each target site also mean that CRISPR-Cas9 gene editing will require expensive clinical trials for each target editing site. The variability in efficiency may also require larger numbers of patients to show treatment efficacy. This would limit the scope of target editing to those which are relatively more common, those in which mutations result ideally in non-coding regions and those diseases which are not amendable to gene therapy, such as autosomal dominant diseases. Another shadow also looms over CRISPR-Cas9 and its use to treat IRDs. Trials using ASOs have also begun and progressed rapidly. ASOs are single-stranded chains of nucleic acids, between 8 and 50 nucleotides in length, which are synthetically generated, that bind to RNA through standard Watson–Crick base pairing. They can prevent translation of a region of mRNA or the translation of mRNA altogether from a mutant allele slowing the progression of a disease. ASOs offer the advantage that they are reversible, have pan-retinal effect from intravitreal injection and appear to have a limited immune response, but may require its continuous delivery every certain time. Three splice-switching ASOs, nusinersen, golodirsen and eteplirsen have already been FDA approved to treat other diseases. The first of the steric block ASO IRD trials, targeting exon 13 of *USH2A* is currently preparing for phase III trial with early limited release of promising preclinical data and early clinical data [NCT03780257] (115). More concerns for the development of CRISPR-Cas9 trials is that ASO trials appear to be targeting the same loci as the early CRISPR-Cas9 trials (26, 116). This not only means that there is direct competition for treatment but may also limit the pool of patients required to complete larger phase III studies in a timely manner for these rare diseases. ASOs however, also show marked variability in specificity at RNA loci. So, it may be that CRISPR-Cas9 is more suitable at some loci and ASOs may be a better tool to treat other diseases at other loci. More human clinical trial data will be required to assist in informing these decisions.

Despite CRISPR-Cas9 gene editing system was discovered less than a decade ago, the system is now widely used by scientists, with a significant impact in the biomedical area. Although its unpredictability presents some challenges for its clinical application, robust safety, and ultimately efficacy data, from the early clinical trials will be essential to support the benefits of its wider implementation. Whether CRISPR-Cas9 will be commonly used to treat not only IRDs, but also other human diseases, is yet to be seen.

## Author Contributions

JH-J wrote, reviewed, and edited the manuscript. GR-U helped write and generate figures and tables. SB initiated the manuscript, helped write, review, and edit the manuscript. All authors contributed to the article and approved the submitted version.

## Funding

SB was supported by a Foundation Fighting Blindness Career Development Award.

## Conflict of Interest

SB is PI at the University of California San Diego for the BRILLLIANCE “Single Ascending Dose Study in Participants with LCA10.” The remaining authors declare that the research was conducted in the absence of any commercial or financial relationships that could be construed as a potential conflict of interest.

## Publisher's Note

All claims expressed in this article are solely those of the authors and do not necessarily represent those of their affiliated organizations, or those of the publisher, the editors and the reviewers. Any product that may be evaluated in this article, or claim that may be made by its manufacturer, is not guaranteed or endorsed by the publisher.

## Supplementary Material

Supplementary Figure 1 DNA repair mechanisms following CRISPR-Cas9 double-strand break. Non-homologous end joining [NHEJ] is the most common form of DNA repair and enables restoration of the double DNA strands. However, it often leads to random insertions or deletions of bases. Less frequently, homology-directed repair [HDR] can be used to repair double-strand breaks. HDR uses a template to help correct the double-strand break. This template can be from the matching alternate allele naturally or from a donor DNA template provided artificially. Homology independent targeted insertion [HITI] strategy introduces two predetermined CRISPR-Cas9 target cleavage sites into donor DNA fragment. After Cas9 cuts the targeted sites in both genomic DNA and donor DNA, the donor fragment undergoes targeted integration by NHEJ repair. The process stops when the fragment is inserted in the desired orientation. *Illustration created with BioRender software*.

Supplementary Figure 2 Gene editing approaches to treat inherited retinal diseases. **(A)** *In vivo* approach by subretinal or intravitreal administration of therapeutic vectors. Vector delivery can be achieved by subretinal injection, which involves the formation of a transient retinal detachment that resolves spontaneously. Intravitreal injection is less invasive and produces fewer iatrogenic complications, but it may deliver therapeutic genes less efficiently. **(B)** *Ex vivo* approaches to gene editing and correction of human induced pluripotent stem cells [iPSCs]. Patient-specific iPSCs can be generated using skin biopsies (or other tissue biopsies) to obtain fibroblasts or other somatic cells that can be reprogrammed using specific “reprogramming transcription factors.” Following iPSC expansion and differentiation into retinal cells (photoreceptors, RPE or laminated retinal tissue), these “corrected” cells can be transplanted back into the patient to repair retinal structures and restore vision. *Illustration created with BioRender software*.

## References

[1] Duncan JL Pierce EA Laster AM Daiger SP Birch DG Ash JD. Inherited retinal degenerations: Current landscape and knowledge gaps. Transl Vis Sci Technol. (2018) 7:6. 10.1167/tvst.7.4.6 30034950 PMC6052953

[2] Cremers FPM Boon CJF Bujakowska K Zeitz C. Special issue introduction: Inherited retinal disease: Novel candidate genes, genotype-phenotype correlations, and inheritance models. Genes. (2018) 9:215. 10.3390/genes9040215 29659558 PMC5924557

[3] Bessant DA Ali RR Bhattacharya SS. Molecular genetics and prospects for therapy of the inherited retinal dystrophies. Curr Opin Genet Dev. (2001) 11:307–16. 10.1016/S0959-437X(00)00195-7 11377968

[4] Sahel JA Marazova K Audo I. Clinical characteristics and current therapies for inherited retinal degenerations. Cold Spring Harb Perspect Med. (2014) 5:a017111. 10.1101/cshperspect.a017111 25324231 PMC4315917

[5] Veleri S Lazar CH Chang B Sieving PA Banin E Swaroop A. Biology and therapy of inherited retinal degenerative disease: Insights from mouse models. Dis Model Mech. (2015) 8:109–29. 10.1242/dmm.017913 25650393 PMC4314777

[6] Smith AJ Carter SP Kennedy BN. Genome editing: the breakthrough technology for inherited retinal disease? Expert Opin Biol Ther. (2017) 17:1245–54. 10.1080/14712598.2017.1347629 28695744

[7] RetNet: Summaries. Available online at: https://sph.uth.edu/retnet/sum-dis.htm (accessed March 10, 2021).

[8] Tiwari A Lemke J Altmueller J Thiele H Glaus E Fleischhauer J. Identification of novel and recurrent disease-causing mutations in retinal dystrophies using whole exome sequencing (WES): benefits and limitations. PLoS ONE. (2016) 11:e0158692. 10.1371/journal.pone.0158692 27391102 PMC4938416

[9] Riera M Navarro R Ruiz-Nogales S Méndez P Burés-Jelstrup A Corcóstegui B. Whole exome sequencing using Ion Proton system enables reliable genetic diagnosis of inherited retinal dystrophies. Sci Rep. (2017) 7:42078. 10.1038/srep42078 28181551 PMC5299602

[10] Beryozkin A Shevah E Kimchi A Mizrahi-Meissonnier L Khateb S Ratnapriya R. Whole exome sequencing reveals mutations in known retinal disease genes in 33 out of 68 israeli families with inherited retinopathies. Sci Rep. (2015) 5:13187. 10.1038/srep13187 26306921 PMC4549705

[11] Zhao L Wang F Wang H Li Y Alexander S Wang K. Next-generation sequencing-based molecular diagnosis of 82 retinitis pigmentosa probands from Northern Ireland. Hum Genet. (2015) 134:217–30. 10.1007/s00439-014-1512-7 25472526 PMC4347882

[12] Samiy N. Gene therapy for retinal diseases. J Ophthalmic Vis Res. (2014) 9:506–9. 10.4103/2008-322X.150831 25709778 PMC4329713

[13] Berger W Kloeckener-Gruissem B Neidhardt J. The molecular basis of human retinal and vitreoretinal diseases. Prog Retin Eye Res. (2010) 29:335–75. 10.1016/j.preteyeres.2010.03.004 20362068

[14] Koulisis N Nagiel A. Precision therapy for inherited retinal disease: at the forefront of genomic medicine. Clin Lab Med. (2020) 40:189–204. 10.1016/j.cll.2020.02.007 32439068

[15] Gemayel MC Bhatwadekar AD Ciulla T. RNA therapeutics for retinal diseases. Expert Opin Biol Ther. (2021) 21:603–13. 10.1080/14712598.2021.1856365 33307874 PMC8087622

[16] Cox DBT Platt RJ Zhang F. Therapeutic genome editing: Prospects and challenges. Nat Med. (2015) 21:121–31. 10.1038/nm.3793 25654603 PMC4492683

[17] Trapani I Auricchio A. Seeing the light after 25 years of retinal gene therapy. Trends Mol Med. (2018) 24:669–81. 10.1016/j.molmed.2018.06.006 29983335

[18] Garafalo AV Cideciyan AV Héon E Sheplock R Pearson A WeiYang Yu C. Progress in treating inherited retinal diseases: Early subretinal gene therapy clinical trials and candidates for future initiatives. Prog Retin Eye Res. (2020) 77:100827. 10.1016/j.preteyeres.2019.100827 31899291 PMC8714059

[19] Timmers AM Newmark JA Turunen HT Farivar T Liu J Song C. Ocular inflammatory response to intravitreal injection of adeno-associated virus vector: relative contribution of genome and capsid. Hum Gene Ther. (2020) 31:80–9. 10.1089/hum.2019.144 31544533

[20] Pierce EA Bennett J. The status of RPE65 gene therapy trials: safety and efficacy. Cold Spring Harb Perspect Med. (2015) 5:1–16. 10.1101/cshperspect.a017285 25635059 PMC4561397

[21] Verrier JD Madorsky I Coggin WE Geesey M Hochman M Walling E. Bicistronic lentiviruses containing a viral 2A cleavage sequence reliably co-express two proteins and restore vision to an animal model of LCA1. PLoS ONE. (2011) 6:e20553. 10.1371/JOURNAL.PONE.0020553 21647387 PMC3103589

[22] Burnight ER Wiley LA Drack A V. Braun TA Anfinson KR Kaalberg EE. CEP290 gene transfer rescues Leber congenital amaurosis cellular phenotype. Gene Ther. (2014) 21:662–72. 10.1038/gt.2014.39 24807808 PMC4188442

[23] Shahi PK Hermans D Sinha D Brar S Moulton H Stulo S. Gene augmentation and readthrough rescue channelopathy in an iPSC-RPE model of congenital blindness. Am J Hum Genet. (2019) 104:310. 10.1016/J.AJHG.2018.12.019 30686507 PMC6369573

[24] Dezawa M Takano M Negishi H Mo X Oshitari T Sawada H. Gene transfer into retinal ganglion cells by in vivo electroporation: a new approach. Micron. (2002) 33:1–6. 10.1016/s0968-4328(01)00002-6 11473808

[25] Kelley RA Conley SM Makkia R Watson JN Han Z Cooper MJ. DNA nanoparticles are safe and nontoxic in non-human primate eyes. Int J Nanomed. (2018) 13:1361–79. 10.2147/IJN.S157000 29563793 PMC5849385

[26] Cideciyan AV Jacobson SG Drack AV Ho AC Charng J Garafalo AV. Effect of an intravitreal antisense oligonucleotide on vision in Leber congenital amaurosis due to a photoreceptor cilium defect. Nat Med. (2019) 25:225–8. 10.1038/S41591-018-0295-0 30559420

[27] Xiong W Wu DM Xue Y Wang SK Chung MJ Ji X. AAV cis -regulatory sequences are correlated with ocular toxicity. Proc Natl Acad Sci USA. (2019) 116:5785–94. 10.1073/pnas.1821000116 30833387 PMC6431174

[28] Vázquez-Domínguez I Garanto A Collin RWJ. Molecular therapies for inherited retinal diseases-current standing, opportunities and challenges. Genes (Basel). (2019) 10:654. 10.3390/genes10090654 31466352 PMC6770110

[29] Jinek M Chylinski K Fonfara I Hauer M Doudna JA Charpentier E. A programmable dual-RNA-guided DNA endonuclease in adaptive bacterial immunity. Science. (2012) 337:816–21. 10.1126/science.1225829 22745249 PMC6286148

[30] Sanjurjo-Soriano C Kalatzis V. Guiding lights in genome editing for inherited retinal disorders: implications for gene and cell therapy. Neural Plast. (2018) 2018:5056279. 10.1155/2018/5056279 29853845 PMC5964415

[31] Hsu PD Scott DA Weinstein JA Ran FA Konermann S Agarwala V. DNA targeting specificity of RNA-guided Cas9 nucleases. Nat Biotechnol. (2013) 31:827–32. 10.1038/nbt.2647 23873081 PMC3969858

[32] Bajan S Hutvagner G. RNA-based therapeutics: from antisense oligonucleotides to miRNAs. Cells. (2020) 9:137. 10.3390/cells9010137 31936122 PMC7016530

[33] Gaj T Gersbach CA Barbas CF. ZFN, TALEN, and CRISPR/Cas-based methods for genome engineering. Trends Biotechnol. (2013) 31:397–405. 10.1016/j.tibtech.2013.04.004 23664777 PMC3694601

[34] Santiago Y Chan E Liu P Orlando S Zhang L Urnov FD. Targeted gene knockout in mammalian cells by using engineered zinc-finger nucleases. Proc Natl Acad Sci USA. (2008) 105:5809–14. 10.1073/pnas.0800940105 18359850 PMC2299223

[35] Brandsma I Gent DC. Pathway choice in DNA double strand break repair: observations of a balancing act. Genome Integr. (2012) 3:9. 10.1186/2041-9414-3-9 23181949 PMC3557175

[36] Lieber MR. The mechanism of double-strand DNA break repair by the nonhomologous DNA end-joining pathway. Annu Rev Biochem. (2010) 79:181–211. 10.1146/annurev.biochem.052308.093131 20192759 PMC3079308

[37] Gragg M Park PSH. Detection of misfolded rhodopsin aggregates in cells by Förster resonance energy transfer. Methods Cell Biol. (2019) 149:87–105. 10.1016/bs.mcb.2018.08.007 30616829 PMC6941733

[38] Latella MC Di Salvo MT Cocchiarella F Benati D Grisendi G Comitato A. *In vivo* editing of the human mutant rhodopsin gene by electroporation of plasmid-based CRISPR/Cas9 in the mouse retina. Mol Ther - Nucleic Acids. (2016) 5:e389. 10.1038/mtna.2016.92 27874856 PMC5155324

[39] Giannelli SG Luoni M Castoldi V Massimino L Cabassi T Angeloni D. Cas9/sgRNA selective targeting of the P23H rhodopsin mutant allele for treating retinitis pigmentosa by intravitreal AAV9.PHP.B-based delivery. Hum Mol Genet. (2018) 27:761–79. 10.1093/hmg/ddx438 29281027

[40] Bakondi B Lv W Lu B Jones MK Tsai Y Kim KJ. *In vivo* CRISPR/Cas9 gene editing corrects retinal dystrophy in the S334ter-3 rat model of autosomal dominant retinitis pigmentosa. Mol Ther. (2016) 24:556–63. 10.1038/mt.2015.220 26666451 PMC4786918

[41] Patrizi C Llado M Benati D Iodice C Marrocco E Guarascio R. Allele-specific editing ameliorates dominant retinitis pigmentosa in a transgenic mouse model. Am J Hum Genet. (2021) 108:295–308. 10.1016/j.ajhg.2021.01.006 33508235 PMC7896132

[42] Suzuki K Tsunekawa Y Hernandez-Benitez R Wu Y Zhu J Kim EJ. In vivo genome editing via CRISPR/Cas9 mediated homology-independent targeted integration. Nature. (2016) 540:144–9. 10.1038/nature20565 27851729 PMC5331785

[43] Suzuki K Belmonte-Izpisua JC. *In vivo* genome editing via the HITI method as a tool for gene therapy. J Hum Genet. (2017) 63:157–64. 10.1038/s10038-017-0352-4 29215090

[44] Nishiyama J Mikuni T Yasuda R. Virus-mediated genome editing via homology-directed repair in mitotic and postmitotic cells in mammalian brain. Neuron. (2017) 96:755–68.e5. 10.1016/j.neuron.2017.10.004 29056297 PMC5691606

[45] Wu W-H Tsai Y-T Justus S Lee T-T Zhang L Lin C-S. CRISPR repair reveals causative mutation in a preclinical model of retinitis pigmentosa. Mol Ther. (2016) 24:1388–94. 10.1038/mt.2016.107 27203441 PMC5023380

[46] Jo DH Song DW Cho CS Kim UG Lee KJ Lee K. CRISPR-Cas9–mediated therapeutic editing of Rpe65 ameliorates the disease phenotypes in a mouse model of Leber congenital amaurosis. Sci Adv. (2019) 5:eaax1210. 10.1126/sciadv.aax1210 31692906 PMC6821465

[47] Wu W Tang L D'Amore PA Lei H. Application of CRISPR-Cas9 in eye disease. Exp Eye Res. (2017) 161:116–23. 10.1016/j.exer.2017.06.007 28619505

[48] Llado Santaeularia M Esposito F Iodice C Marrocco E Auricchio A. Homology-independent targeted integration in photoreceptors. ARVO Annual Meeting Abstract. Investig Ophtalmol Vis Sci. (2019) 60:4228.

[49] Komor AC Kim YB Packer MS Zuris JA Liu DR. Programmable editing of a target base in genomic DNA without double-stranded DNA cleavage. Nature. (2016) 533:420–4. 10.1038/nature17946 27096365 PMC4873371

[50] Gaudelli NM Komor AC Rees HA Packer MS Badran AH Bryson DI. Publisher Correction: Programmable base editing of A^*^T to G^*^C in genomic DNA without DNA cleavage. Nature. (2018) 559:E8. 10.1038/s41586-018-0070-x 29720650

[51] Kim YB Komor AC Levy JM Packer MS Zhao KT Liu DR. Increasing the genome-targeting scope and precision of base editing with engineered Cas9-cytidine deaminase fusions. Nat Biotechnol. (2017) 35:371–6. 10.1038/nbt.3803 28191901 PMC5388574

[52] Anzalone A V Randolph PB Davis JR Sousa AA Koblan LW Levy JM. Search-and-replace genome editing without double-strand breaks or donor DNA. Nature. (2019) 576:149–57. 10.1038/s41586-019-1711-4 31634902 PMC6907074

[53] Larson MH Gilbert LA Wang X Lim WA Weissman JS Qi LS. CRISPR interference (CRISPRi) for sequence-specific control of gene expression. Nat Protoc. (2013) 8:2180–96. 10.1038/nprot.2013.132 24136345 PMC3922765

[54] Gilbert LA Larson MH Morsut L Liu Z Brar GA Torres SE. CRISPR-mediated modular RNA-guided regulation of transcription in eukaryotes. Cell. (2013) 154:442–51. 10.1016/j.cell.2013.06.044 23849981 PMC3770145

[55] Pulecio J Verma N Mejía-Ramírez E Huangfu D Raya A. CRISPR/Cas9-based engineering of the epigenome. Cell Stem Cell. (2017) 21:431–47. 10.1016/j.stem.2017.09.006 28985525 PMC6205890

[56] Qi LS Larson MH Gilbert LA Doudna JA Weissman JS Arkin AP. Repurposing CRISPR as an RNA-guided platform for sequence-specific control of gene expression. Cell. (2013) 152:1173–83. 10.1016/j.cell.2013.02.022 23452860 PMC3664290

[57] Thakore PI D'Ippolito AM Song L Safi A Shivakumar NK Kabadi AM. Highly specific epigenome editing by CRISPR-Cas9 repressors for silencing of distal regulatory elements. Nat Methods. (2015) 12:1143–9. 10.1038/nmeth.3630 26501517 PMC4666778

[58] Moreno AM Fu X Zhu J Katrekar D Shih Y-R V Marlett J. *In situ* gene therapy via AAV-CRISPR-Cas9-mediated targeted gene regulation. Mol Ther. (2018) 26:1818–27. 10.1016/j.ymthe.2018.04.017 29754775 PMC6035733

[59] Gilbert LA Horlbeck MA Adamson B Villalta JE Chen Y Whitehead EH. Genome-scale CRISPR-mediated control of gene repression and activation. Cell. (2014) 159:647–61. 10.1016/j.cell.2014.09.029 25307932 PMC4253859

[60] Cox DBT Gootenberg JS Abudayyeh OO Franklin B Kellner MJ Joung J. RNA editing with CRISPR-Cas13. Science. (2017) 358:1019–27. 10.1126/science.aaq0180 29070703 PMC5793859

[61] Schmelas C Grimm D. Split Cas9, not hairs - advancing the therapeutic index of CRISPR technology. Biotechnol J. (2018) 13:e1700432. 10.1002/biot.201700432 29316283

[62] Goswami R Subramanian G Silayeva L Newkirk I Doctor D Chawla K. Gene therapy leaves a vicious cycle. Front Oncol. (2019) 9:297. 10.3389/fonc.2019.00297 31069169 PMC6491712

[63] Dismuke DJ Tenenbaum L Samulski RJ. Biosafety of recombinant adeno-associated virus vectors. Curr Gene Ther. (2013) 13:434–52. 10.2174/15665232113136660007 24195602

[64] Marangoni D Wu Z Wiley HE Zeiss CJ Vijayasarathy C Zeng Y. Preclinical safety evaluation of a recombinant AAV8 vector for X-linked retinoschisis after intravitreal administration in rabbits. Hum Gene Ther Clin Dev. (2014) 25:202–11. 10.1089/humc.2014.067 25211193 PMC4275775

[65] Charlesworth CT Deshpande PS Dever DP Camarena J Lemgart VT Cromer MK. Identification of preexisting adaptive immunity to Cas9 proteins in humans. Nat Med. (2019) 25:249–54. 10.1038/s41591-018-0326-x 30692695 PMC7199589

[66] Li A Tanner MR Lee CM Hurley AE De Giorgi M Jarrett KE. AAV-CRISPR gene editing is negated by pre-existing immunity to Cas9. Mol Ther. (2020) 28:1432–41. 10.1016/j.ymthe.2020.04.017 32348718 PMC7264438

[67] Ruan G-X Barry E Yu D Lukason M Cheng SH Scaria A. CRISPR/Cas9-mediated genome editing as a therapeutic approach for leber congenital amaurosis 10. Mol Ther. (2017) 25:331–41. 10.1016/j.ymthe.2016.12.006 28109959 PMC5368591

[68] Huang X Zhou G Wu W Duan Y Ma G Song J. Genome editing abrogates angiogenesis *in vivo*. Nat Commun. (2017) 8:112. 10.1038/s41467-017-00140-3 28740073 PMC5524639

[69] Kim K Park SW Kim JH Lee SH Kim D Koo T. Genome surgery using Cas9 ribonucleoproteins for the treatment of age-related macular degeneration. Genome Res. (2017) 27:419–26. 10.1101/gr.219089.116 28209587 PMC5340969

[70] Li Q Miller R Han P Pang J Dinculescu A Chiodo V. Intraocular route of AAV2 vector administration defines humoral immune response and therapeutic potential. Mol Vis. (2008) 14:1760–9. 18836574 PMC2559816

[71] Li L Hu S Chen X. Non-viral delivery systems for CRISPR/Cas9-based genome editing: Challenges and opportunities. Biomaterials. (2018) 171:207–18. 10.1016/j.biomaterials.2018.04.031 29704747 PMC5944364

[72] Zuris JA Thompson DB Shu Y Guilinger JP Bessen JL Hu JH. Cationic lipid-mediated delivery of proteins enables efficient protein-based genome editing *in vitro* and *in vivo*. Nat Biotechnol. (2015) 33:73–80. 10.1038/nbt.3081 25357182 PMC4289409

[73] Li L Song L Liu X Yang X Li X He T. Artificial virus delivers CRISPR-Cas9 system for genome editing of cells in mice. ACS Nano. (2017) 11:95–111. 10.1021/acsnano.6b04261 28114767

[74] Zhou W Cui H Ying L Yu XF. Enhanced cytosolic delivery and release of CRISPR/Cas9 by black phosphorus nanosheets for genome editing. Angew Chem Int Ed Engl. (2018) 57:10268–72. 10.1002/anie.201806941 29939484

[75] Lee B Lee K Panda S Gonzales-Rojas R Chong A Bugay V. Nanoparticle delivery of CRISPR into the brain rescues a mouse model of fragile X syndrome from exaggerated repetitive behaviours. Nat Biomed Eng. (2018) 2:497–507. 10.1038/s41551-018-0252-8 30948824 PMC6544395

[76] Gao X Tao Y Lamas V Huang M Yeh WH Pan B. Treatment of autosomal dominant hearing loss by *in vivo* delivery of genome editing agents. Nature. (2018) 553:217–21. 10.1038/nature25164 29258297 PMC5784267

[77] Mora-Raimundo P Lozano D Manzano M Vallet-Regi M. Nanoparticles to knockdown osteoporosis-related gene and promote osteogenic marker expression for osteoporosis treatment. ACS Nano. (2019) 13:5451–64. 10.1021/acsnano.9b00241 31071265 PMC6588271

[78] Lee K Conboy M Park HM Jiang F Kim HJ Dewitt MA. Nanoparticle delivery of Cas9 ribonucleoprotein and donor DNA in vivo induces homology-directed DNA repair. Nat Biomed Eng. (2017) 1:889–901. 10.1038/s41551-017-0137-2 29805845 PMC5968829

[79] Wang HX Song Z Lao YH Xu X Gong J Cheng D. Nonviral gene editing via CRISPR/Cas9 delivery by membrane-disruptive and endosomolytic helical polypeptide. Proc Natl Acad Sci USA. (2018) 115:4903–8. 10.1073/pnas.1712963115 29686087 PMC5948953

[80] Chou S-J Yang P Ban Q Yang Y-P Wang M-L Chien C-S. Dual supramolecular nanoparticle vectors enable CRISPR/Cas9-mediated knockin of retinoschisin 1 gene—a potential nonviral therapeutic solution for X-linked juvenile retinoschisis. Adv Sci. (2020) 23:1903432. 10.1002/advs.201903432 32440478 PMC7237855

[81] Wang D Zhang F Gao G. CRISPR-based therapeutic genome editing: strategies and *in vivo* delivery by AAV vectors. Cell. (2020) 181:136–50. 10.1016/j.cell.2020.03.023 32243786 PMC7236621

[82] Maeder ML Stefanidakis M Wilson CJ Baral R Barrera LA Bounoutas GS. Development of a gene-editing approach to restore vision loss in Leber congenital amaurosis type 10. Nat Med. (2019) 25:229–33. 10.1038/s41591-018-0327-9 30664785

[83] Ran FA Cong L Yan WX Scott DA Gootenberg JS Kriz AJ. *In vivo* genome editing using Staphylococcus aureus Cas9. Nature. (2015) 520:186–91. 10.1038/nature14299 25830891 PMC4393360

[84] Zetsche B Volz SE Zhang F. A split-Cas9 architecture for inducible genome editing and transcription modulation. Nat Biotechnol. (2015) 33:139–42. 10.1038/nbt.3149 25643054 PMC4503468

[85] Wright A V Sternberg SH Taylor DW Staahl BT Bardales JA Kornfeld JE. Rational design of a split-Cas9 enzyme complex. Proc Natl Acad Sci USA. (2015) 112:2984–9. 10.1073/pnas.1501698112 25713377 PMC4364227

[86] Kim E Koo T Park SW Kim D Kim K Cho HY. In vivo genome editing with a small Cas9 orthologue derived from *Campylobacter jejuni*. Nat Commun. (2017) 8:14500. 10.1038/ncomms14500 28220790 PMC5473640

[87] Kaminski R Bella R Yin C Otte J Ferrante P Gendelman HE. Excision of HIV-1 DNA by gene editing: A proof-of-concept in vivo study. Gene Ther. (2016) 23:690–5. 10.1038/gt.2016.41 27194423 PMC4974122

[88] Kleinstiver BP Prew MS Tsai SQ Nguyen NT Topkar V V Zheng Z. Broadening the targeting range of *Staphylococcus aureus* CRISPR-Cas9 by modifying PAM recognition. Nat Biotechnol. (2015) 33:1293–8. 10.1038/nbt.3404 26524662 PMC4689141

[89] Legut M Daniloski Z Xue X Mckenzie D Guo X Wessels H-H. High-throughput screens of PAM-flexible Cas9 variants for gene knockout and transcriptional modulation. Cell Rep. (2020) 30:2859–68.e5. 10.1016/j.celrep.2020.02.010 32130891 PMC7558435

[90] O'Geen H Yu AS Segal DJ. How specific is CRISPR/Cas9 really? Curr Opin Chem Biol. (2015) 29:72–8. 10.1016/j.cbpa.2015.10.001 26517564 PMC4684463

[91] Guo M Ren K Zhu Y Tang Z Wang Y Zhang B. Structural insights into a high fidelity variant of SpCas9. Cell Res. (2019) 29:183–92. 10.1038/s41422-018-0131-6 30664728 PMC6460432

[92] Slaymaker IM Gao L Zetsche B Scott DA Yan WX Zhang F. Rationally engineered Cas9 nucleases with improved specificity. Science. (2016) 351:84–8. 10.1126/science.aad5227 26628643 PMC4714946

[93] Kleinstiver BP Pattanayak V Prew MS Tsai SQ Nguyen NT Zheng Z. High-fidelity CRISPR–Cas9 nucleases with no detectable genome-wide off-target effects. Nature. (2016) 529:490–5. 10.1038/nature16526 26735016 PMC4851738

[94] Chen JS Dagdas YS Benjamin P Welch MM Sousa AA Harrington LB. Enhanced proofreading governs CRISPR–Cas9 targeting accuracy. Nature. (2017) 550:407–10. 10.1038/nature24268 28931002 PMC5918688

[95] Lee JK Jeong E Lee J Jung M Shin E Kim Y. Directed evolution of CRISPR-Cas9 to increase its specificity. Nat Commun. (2018) 9:3048. 10.1038/s41467-018-05477-x 30082838 PMC6078992

[96] Cong L Ran FA Cox D Lin S Barreto R Habib N. Multiplex genome engineering using CRISPR/Cas systems. Science. (2013) 339:819–23. 10.1126/science.1231143 23287718 PMC3795411

[97] Ran FA Hsu PD Lin C-Y Gootenberg JS Konermann S Trevino AE. Double nicking by RNA-guided CRISPR Cas9 for enhanced genome editing specificity. Cell. (2013) 154:1380–9. 10.1016/j.cell.2013.08.021 23992846 PMC3856256

[98] Chen Y Liu X Zhang Y Wang H Ying H Liu M. A self-restricted CRISPR system to reduce off-target effects. Mol Ther. (2016) 24:1508–10. 10.1038/mt.2016.172 27687135 PMC5113117

[99] Li F Hung SSC Mohd Khalid MKN Wang J-HH Chrysostomou V Wong VHYY. Utility of self-destructing CRISPR/Cas constructs for targeted gene editing in the retina. Hum Gene Ther. (2019) 30:1349–60. 10.1089/hum.2019.021 31373227

[100] Wang H Lu H Lei Y shou Gong C Chen Z Luan Y qiao. Development of a self-restricting CRISPR-Cas9 system to reduce off-target effects. Mol Ther - Methods Clin Dev. (2020) 18:390–401. 10.1016/j.omtm.2020.06.012 32695841 PMC7358219

[101] Canaj H Hussmann JA Li H Beckman KA Goodrich L Cho NH. Deep profiling reveals substantial heterogeneity of integration outcomes in CRISPR knock-in experiments. bioRxiv [preprint]. (2019). 10.1101/841098

[102] Brunner E Yagi R Debrunner M Beck-Schneider D Burger A Escher E. CRISPR-induced double-strand breaks trigger recombination between homologous chromosome arms. Life Sci Alliance. (2019) 2:1–11. 10.26508/lsa.201800267 31196871 PMC6587125

[103] Collin RW Den Hollander AI Der Velde-Visser SDV Bennicelli J Bennett J Cremers FP. Antisense oligonucleotide (AON)-based therapy for Leber congenital amaurosis caused by a frequent mutation in CEP290. Mol Ther - Nucleic Acids. (2012) 1:e14. 10.1038/mtna.2012.3 23343883 PMC3381589

[104] Craige B Tsao CC Diener DR Hou Y Lechtreck KF Rosenbaum JL. CEP290 tethers flagellar transition zone microtubules to the membrane and regulates flagellar protein content. J Cell Biol. (2010) 190:927–40. 10.1083/jcb.201006105 20819941 PMC2935561

[105] Drivas TG Holzbaur ELF Bennett J. Disruption of CEP290 microtubule/membrane-binding domains causes retinal degeneration. J Clin Invest. (2013) 123:4525–39. 10.1172/JCI69448 24051377 PMC3784542

[106] Cideciyan A V Aleman TS Jacobson SG Khanna H Sumaroka A Aguirre GK. Centrosomal-ciliary gene CEP290/NPHP6 mutations result in blindness with unexpected sparing of photoreceptors and visual brain: implications for therapy of Leber congenital amaurosis. Hum Mutat. (2007) 28:1074–83. 10.1002/humu.20565 17554762

[107] Gerard X Perrault I Hanein S Silva E Bigot K Defoort-Delhemmes S. AON-mediated exon skipping restores ciliation in fibroblasts harboring the common leber congenital amaurosis CEP290 mutation. Mol Ther - Nucleic Acids. (2012) 1:e29. 10.1038/mtna.2012.21 23344081 PMC3390222

[108] Garanto A Van Beersum SEC Peters TA Roepman R Cremers FPM Collin RWJ. Unexpected CEP290 mRNA splicing in a humanized knock-in mouse model for Leber congenital amaurosis. PLoS ONE. (2013) 8:e79369. 10.1371/journal.pone.0079369 24223178 PMC3819269

[109] Geller AM Sieving PA. Assessment of foveal cone photoreceptors in Stargardt's macular dystrophy using a small dot detection task. Vision Res. (1993) 33:1509–24. 10.1016/0042-6989(93)90144-l 8351823

[110] Carter-Dawson LD Lavail MM. Rods and cones in the mouse retina. J Comp Neurol. (1979) 188:245–62. 10.1002/cne.901880204 500858

[111] Petrus-Reurer S Winblad N Kumar P Gorchs L Chrobok M Wagner AK. Generation of retinal pigment epithelial cells derived from human embryonic stem cells lacking human leukocyte antigen class I and II. Stem Cell Rep. (2020) 14:648–62. 10.1016/j.stemcr.2020.02.006 32197113 PMC7160308

[112] Editas Medicine Reports On Recent Progress At JP. Morgan Healthcare Conference. (2020). Available online at: https://ir.editasmedicine.com/static-files/03350a40-33d7-413c-b2d6-c46f94ab273f (accessed March 2, 2021).

[113] Mukherjee S Mehta A Ciulla D Bochicchio J Giannoukos G Marco E. In vivo Proof of Concept for EDIT-102 : A CRISPR / Cas9-Based Experimental Medicine for USH2A - Related Inherited Retinal Degeneration Caused by Mutations in Exon 13. In: Presented at the 23rd ASGCT Annual Meeting. Boston, MA (2020).

[114] French LS Mellough CB Chen FK Carvalho LS. A review of gene, drug and cell-based therapies for usher syndrome. Front Cell Neurosci. (2020) 14:183. 10.3389/fncel.2020.00183 32733204 PMC7363968

[115] Dulla K Slijkerman R Diepen HC Van Albert S Dona M Beumer W. Antisense oligonucleotide-based treatment of retinitis pigmentosa caused by USH2A exon 13 mutations. Mol Ther. (2021) 29:1–15. 10.1016/j.ymthe.2021.04.024 33895329 PMC8353187

[116] Panagiotopoulos A Karguth N Pavlou M Böhm S Gasparoni G Walter J. Antisense oligonucleotide- and CRISPR- Cas9-mediated rescue of mRNA splicing for a deep intronic CLRN1 mutation. Mol Ther Nucleic Acid. (2020) 21:1050–61. 10.1016/j.omtn.2020.07.036 32841912 PMC7452116

