# Supplementary figures and images for: Toward the Treatment of Inherited Diseases of the Retina Using CRISPR-Based Gene Editing
Source: Front Med (Lausanne). 2021 Oct 1;8:698521. doi: 10.3389/fmed.2021.698521 (PMC8517184; doi:10.3389/fmed.2021.698521)

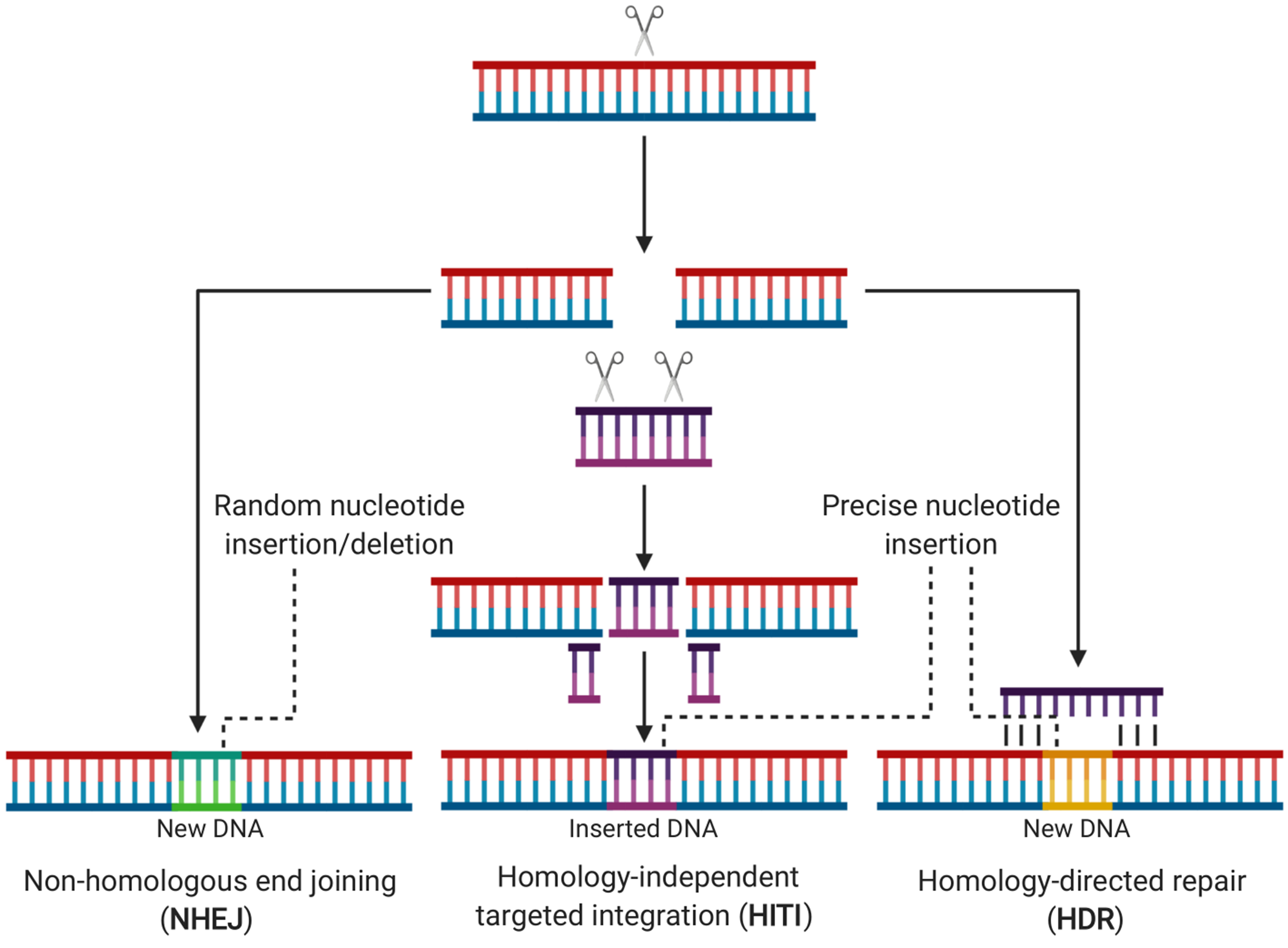

Supplement: Supplementary Figure 1 — DNA repair mechanisms following CRISPR-Cas9 double-strand break. Non-homologous end joining [NHEJ] is the most common form of DNA repair and enables restoration of the double DNA strands. However, it often leads to random insertions or deletions of bases. Less frequently, homology-directed repair [HDR] can be used to repair double-strand breaks. HDR uses a template to help correct the double-strand break. This template can be from the matching alternate allele naturally or from a donor DNA template provided artificially. Homology independent targeted insertion [HITI] strategy introduces two predetermined CRISPR-Cas9 target cleavage sites into donor DNA fragment. After Cas9 cuts the targeted sites in both genomic DNA and donor DNA, the donor fragment undergoes targeted integration by NHEJ repair. The process stops when the fragment is inserted in the desired orientation. Illustration created with BioRender software. [file Image_1.TIFF]

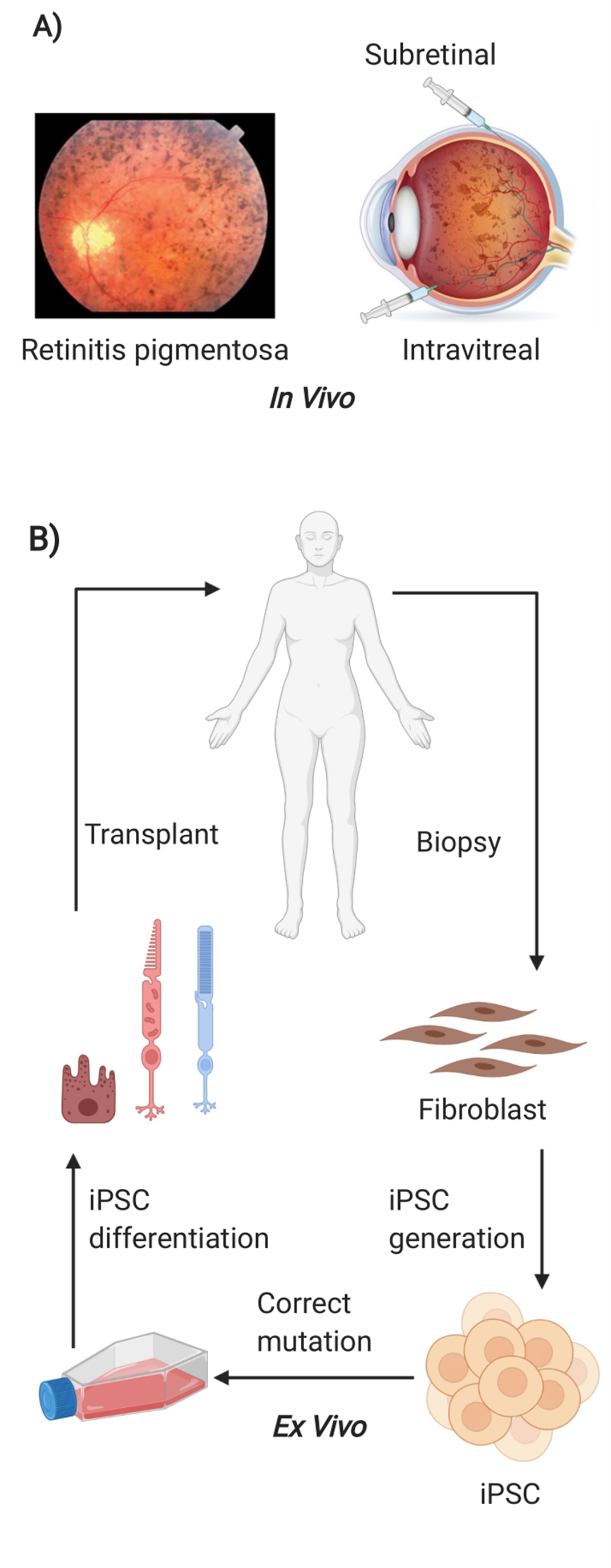

Supplement: Supplementary Figure 2 — Gene editing approaches to treat inherited retinal diseases. (A) In vivo approach by subretinal or intravitreal administration of therapeutic vectors. Vector delivery can be achieved by subretinal injection, which involves the formation of a transient retinal detachment that resolves spontaneously. Intravitreal injection is less invasive and produces fewer iatrogenic complications, but it may deliver therapeutic genes less efficiently. (B) Ex vivo approaches to gene editing and correction of human induced pluripotent stem cells [iPSCs]. Patient-specific iPSCs can be generated using skin biopsies (or other tissue biopsies) to obtain fibroblasts or other somatic cells that can be reprogrammed using specific “reprogramming transcription factors.” Following iPSC expansion and differentiation into retinal cells (photoreceptors, RPE or laminated retinal tissue), these “corrected” cells can be transplanted back into the patient to repair retinal structures and restore vision. Illustration created with BioRender software. [file Image_2.TIFF]
